# Supplementary figures and images for: miRGTF-net: Integrative miRNA-gene-TF network analysis reveals key drivers of breast cancer recurrence
Source: PLoS One. 2021 Apr 14;16(4):e0249424. doi: 10.1371/journal.pone.0249424 (PMC8046230; doi:10.1371/journal.pone.0249424)

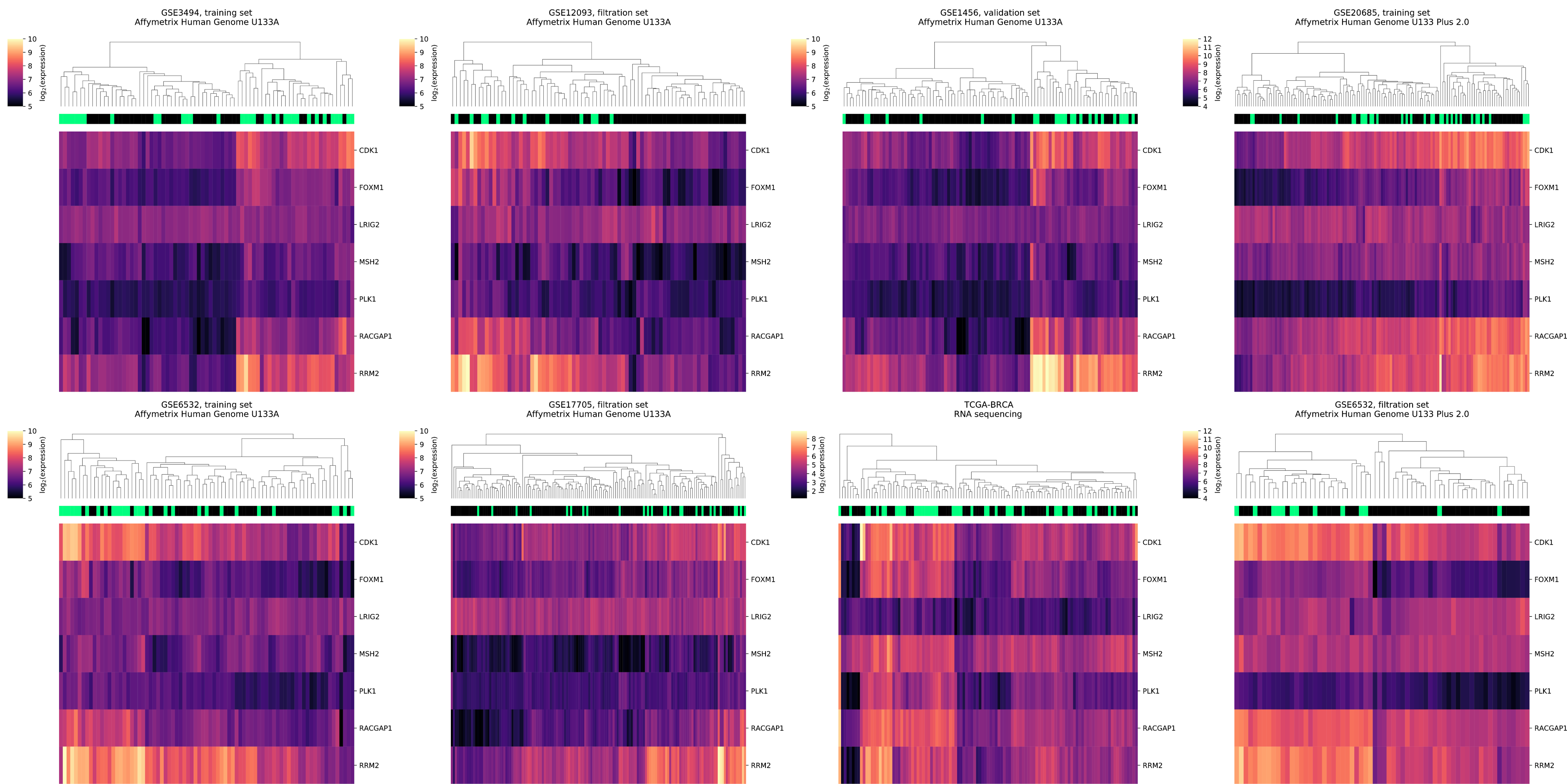

Supplement: S1 Fig — Green color represents patients with recurrence. (TIF) [file pone.0249424.s001.tif]
